# Supplementary material for: Splice-Junction-Based Mapping of Alternative Isoforms in the Human Proteome
Source: Cell Rep. Author manuscript; Available in PMC 2020 Jan 15. (PMC6961840; doi:10.1016/j.celrep.2019.11.026)
Supplement: 2 [file NIHMS1546469-supplement-2.zip › DF1/PXD009737/TestisLysC/TestisLysC_9_RPS15_HGRPGIGATHSSRFIPLR.pdf]

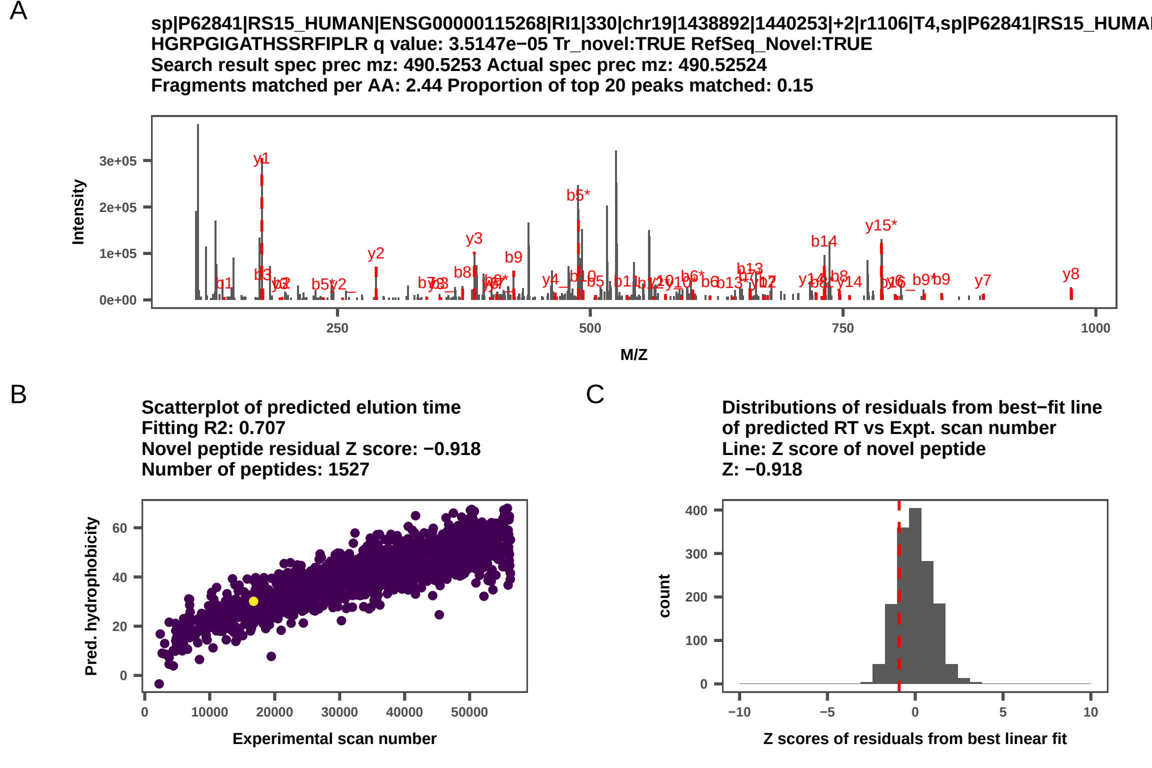
A
sp|P62841|RS15_HUMAN|ENSG00000115268|RI1|330|chr19|1438892|1440253|+2|r1106|T4,sp|P62841|RS15_HUMA
HGRPGIGATHSSRFIPLR q value: 3.5147e−05 Tr_novel:TRUE RefSeq_Novel:TRUE
Search result spec prec mz: 490.5253 Actual spec prec mz: 490.52524
Fragments matched per AA: 2.44 Proportion of top 20 peaks matched: 0.15
Intensity
3e+05
2e+05
1e+05
0e+00
y1
b5*
y15*
b14
y3
y2
b9
b8
y8
y7
M/Z
250
500
750
1000
B
Scatterplot of predicted elution time
Fitting R2: 0.707
Novel peptide residual Z score: −0.918
Number of peptides: 1527
Pred. hydrophobicity
60
40
20
0
0
10000
20000
30000
40000
50000
Experimental scan number
C
Distributions of residuals from best−fit line
of predicted RT vs Expt. scan number
Line: Z score of novel peptide
Z: −0.918
count
400
300
200
100
0
−10
−5
0
5
10
Z scores of residuals from best linear fit
